# Hand Hygiene Practices Among Adults with Diabetes Living in Communities: The 2015 Korea Community Health Survey

**DOI:** 10.3390/ijerph16071279

**Published:** 2019-04-10

**Authors:** Mi Ah Han

**Affiliations:** Department of Preventive Medicine, College of Medicine, Chosun University, 309 Pilmun-Daero, Dong-Gu, Gwangju 61452, Korea; mahan@chosun.ac.kr; Tel.: +82-62-230-6481

**Keywords:** community health centers, diabetes mellitus, hand disinfection, hand hygiene, health surveys

## Abstract

Hand hygiene is the most effective strategy to prevent infectious diseases. This study investigated and compared the hand hygiene practices of adults with diabetes and an age- and gender-matched comparison group. Adults with diabetes (*n* = 22,920) who participated in the 2015 Korea Community Health Survey and an appropriate comparison group (*n* = 22,920) were selected. Descriptive analyses, chi-square tests, and multiple logistic regression analyses were used. Greater than 98% of participants with diabetes recognized that hand hygiene helps prevent infectious diseases. Among participants with diabetes, 84.3%, 82.4%, 72.5%, and 64.1% washed their hands frequently before eating, after using the restroom, after returning from the outdoors, and with soap or hand sanitizer, respectively, but these values were significantly lower than those of the comparison group. After performing multiple regression analyses, treatments for diabetes, being educated about diabetes management and handwashing, and awareness of hemoglobin A1c levels were significantly associated with hand hygiene practices in participants with diabetes. Almost all people with diabetes were aware of the efficacy of hand hygiene, but handwashing rates were significantly lower in people with diabetes than in the comparison group. Considering treatments for diabetes, educational campaigns regarding handwashing, and increasing awareness of handwashing efficacy will help improve hand hygiene in people with diabetes.

## 1. Introduction

Diabetes mellitus is one of the most important public health issues worldwide [1]. The prevalence of diabetes has steadily increased in Korea. The age-standardized prevalence of diabetes was reported to be 9.9%, 10.7%, 10.4%, and 11.4% in men and 7.9%, 8.5%, 8.3%, and 8.7% in women from the Korea National Health and Nutrition Examination Surveys III to VI [2]. 

Because diabetes is a chronic, progressive disease, appropriate self-care as well as clinical treatment are important to increase survival and health quality in patients with diabetes. Self-care behaviors are positively correlated with good glycemic control, reduction of complications, and improvements in quality of life [3,4]. People with diabetes are vulnerable to infectious diseases due to their hyperglycemic condition, which favors immune dysfunction [5]; previous studies have reported a higher risk of infectious diseases among individuals with diabetes [6,7,8]. Thus, maintaining good hygiene is a critical part of self-care in people with diabetes.

Community-dwelling people with diabetes must check their own blood glucose levels and administer insulin themselves. International guidelines recommend that measurements of blood glucose levels and insulin injections should be performed after handwashing with soap and water to acquire a reliable glucose level [9,10]. General hand hygiene, such as handwashing before eating and after using the restroom, effectively prevents infectious diseases, which are common complications of diabetes [11,12,13]. Thus, hand hygiene is important to properly manage diabetes and prevent its complications. 

Previous descriptive studies of outpatients with diabetes reported that self-care activities including personal hygiene were insufficient [14], hygiene behavior was less than ideal, and proper health education provided by health professionals was needed [15]. However, data regarding hand hygiene practices among people with diabetes living in communities are limited. The Korea Community Health Survey (KCHS), conducted annually since 2008, produces community-level health statistics to establish and evaluate the community health care plan. The purpose of this study was to investigate and compare the hand hygiene practices of people with diabetes living in communities in Korea with a comparison group without diabetes. Factors associated with hand hygiene practices among people with diabetes were also investigated using nationwide, community-based surveys.

## 2. Materials and Methods

### 2.1. Data Source and Study Population

The 2015 KCHS was based on 254 communities and conducted by the Korea Centers for Disease Prevention and Control, 17 metropolitan cities, 254 community health centers, and 35 community universities and committees. Stratified cluster sampling methods were used to select sample areas, and a systematic sampling method was used to select sample households. Trained health interviewers visited the selected households, and all household members older than 19 years of age in the sample household were surveyed using computer-assisted personal interviewing. Informed consent was obtained from all individual participants included in the study. Detailed information about data collection is provided elsewhere [16,17]. 

Individuals with diabetes were defined as providing a “yes” answer to the question: “Have you ever been diagnosed with diabetes by a physician?” An exact age- and gender-matched comparison group was selected among participants without a history of diabetes. A total of 228,558 adults participated in the 2015 KCHS, and 22,937 participants were identified as people with diabetes. After excluding 17 participants who had no data regarding hand hygiene practices, the final number of the study population included 22,920 people with diabetes and 22,920 people in the comparison group.

### 2.2. Hand Hygiene Practices

Awareness of handwashing efficacy was defined as answers of “very helpful” or “helpful” to the question: “How do you think handwashing helps prevent infectious disease?” (available answers: very helpful, helpful, not helpful, or not helpful at all). Frequent handwashing before eating, after using the restroom, and after returning from the outdoors were defined as answers of “always” or “often” for each situation to the question: “How frequently did you wash your hands during the last week?” (available answers: always, often, sometimes, or rarely). Frequent handwashing with soap or hand sanitizer was defined as answers of “always” or “often” to the question: “How frequently did you use soap or hand sanitizer when you wash your hands?” (available answers: always, often, sometimes, rarely, or never). Exposure to education and public relation campaigns about handwashing was defined as an answer of “yes” to the question: “Were you educated or exposed to a public relations campaign for handwashing during the last year?”

### 2.3. Covariates

The following general characteristics were assessed for each study participant: gender (male, female), age (19–44, 45–64, or ≥65 years), marital status (single, with spouse, or divorced/separated/widowed), education (uneducated, elementary school, middle school, high school, or ≥ college), smoking (never, former, or current), alcohol drinking frequency (none, ≤1 drink/month, or ≥2 drinks/month), and disease history (yes, no). Disease history included hypertension, dyslipidemia, arthritis, asthma, rhinitis, and atopic dermatitis.

For people with diabetes, the following diabetes-related characteristics were also assessed: treatments for diabetes were classified as insulin, an oral hypoglycemic agent, or none (including nonpharmacological methods, such as exercise and diet). Educational exposure was defined as education in a medical clinic, oriental medical clinic, or community health center for diabetes management (excluding consultation with healthcare providers for fewer than 10 minutes during a typical appointment). Awareness of hemoglobin A1c (HbA1c) level was also investigated (yes or no); HbA1c is used to measure how well diabetes is controlled because it reflects average blood sugar levels over the last three months. Examination of fundoscopy for diabetic eye complications or a urine test (microalbuminuria) for kidney complications during the previous year were investigated.

### 2.4. Statistical Analysis

Comparisons of general characteristics, as well as numbers and proportions of hand hygiene practices, between people with diabetes and the age- and gender-matched comparison group were calculated and examined using chi-square tests. Multiple logistic regressions were performed to assess whether hand hygiene practices significantly differed from -comparison group after adjusting the covariates. Factors associated with frequent handwashing were investigated using multiple logistic regression analyses in people with diabetes. Statistical analyses were performed using SAS version 9.4 (SAS Institute, Cary, NC, USA), and *p* values less than 0.05 were considered statistically significant.

## 3. Results

Among all the study population, 48.6% were male and 56.5% were older than 65 years of age. Marital status, education level, smoking status, alcohol drinking frequency, and disease history significantly differed between people with and without diabetes. Approximately 12.0% of people with diabetes were not undergoing treatment for diabetes, and 80.0% and 8.0% were treated with an oral hypoglycemic agent and insulin, respectively. Additionally, 27.9% were educated about managing their diabetes, and 26.7% were aware of their HbA1c level; fewer than half (47.7%) were examined for diabetes complications during the past year (Table 1).

Greater than 98% of participants were aware of handwashing efficacy, with no difference between people with and without diabetes. Frequent handwashing before eating [84.3% vs. 86.1%, adjusted odds ratio (aOR) = 0.87, 95% confidence interval (CI) = 0.83–0.92], after using the restroom (82.4% vs. 83.9%, aOR = 0.90, 95% CI = 0.85–0.95), after returning from the outdoors (72.5% vs. 74.3%, aOR = 0.91, 95% CI = 0.87–0.96), and with soap or hand sanitizer (64.1% vs. 65.2%, aOR = 0.94, 95% CI = 0.91–0.98) was significantly lower in people with diabetes than in people without diabetes. The proportion of those exposed to educational and public relations campaigns about handwashing was also lower in people with diabetes than in the comparison group (68.7% vs. 69.6%, aOR = 0.95, 95% CI = 0.91–0.99) (Table 2).

Females with diabetes were more likely to wash their hands frequently before eating (aOR = 3.21, 95% CI = 2.83–3.65), after using the restroom (aOR = 2.86, 95% CI = 2.53–3.23), after returning from the outdoors (aOR = 1.63, 95% CI = 1.47–1.81), and with soap or hand sanitizer (aOR = 1.24, 95% CI = 1.13–1.37) compared to males with diabetes after multiple logistic regression analyses. A higher education level was associated with a higher aOR for frequent handwashing. People with diabetes who were either not treated or treated with an oral hypoglycemic agent more frequently washed their hands with soap or hand sanitizer (none: aOR = 1.33, 95% CI = 1.16–1.52; oral hypoglycemic agent: aOR = 1.12, 95% CI = 1.01–1.25) after returning from the outdoors (none: aOR = 1.36, 95% CI = 1.18–1.58; oral hypoglycemic agent: aOR = 1.15, 95% CI = 1.02–1.28) compared to those treated with insulin. Those who were educated about diabetes management, aware of their HbA1c level, examined for complications during the past year, and exposed to educational and public relations campaigns about handwashing had a significantly higher aOR for frequent handwashing (Table 3).

## 4. Discussion

Hand hygiene is one of the best ways to prevent infectious diseases [18]. This study investigated and compared hand hygiene practices among people with diabetes living in communities and a comparison group using data from the 2015 KCHS. Almost all study participants recognized that handwashing is an effective way to prevent infectious diseases. Although awareness of handwashing efficacy between people with and without diabetes did not differ, frequent handwashing rates were significantly lower in people with diabetes compared to an age- and gender-matched comparison group. Fewer than two-thirds of people washed their hands with soap or hand sanitizer. The U.S. Centers for Disease Control and Prevention recommends handwashing with soap and clean running water to prevent the spread of disease [19]. A randomized controlled trial reported that handwashing with soap more effectively reduced bacterial load compared to water alone [20]. Thus, the importance of handwashing with soap must be conveyed to people with diabetes, because these individuals have a higher risk of infectious disease [21]. Unfortunately, this study could not distinguish the location of handwashing, so future studies of the environment in which handwashing occurs and the supply of soap and hand sanitizer in public areas are needed.

Hand hygiene practices were significantly lower in people with diabetes treated with insulin compared to untreated people and those treated with an oral hypoglycemic agent. A previous cross-sectional study described self-administration techniques for insulin in patients with diabetes mellitus; these authors reported that 88.8% of participants used water and soap or liquid detergent before preparing and administering the insulin [22]. A tertiary care center study reported that handwashing was practiced by only 70% of patients with diabetes, with a significant gap between insulin administration guidelines and current insulin injection practices [23,24]. Additionally, self-monitoring of blood glucose levels is an important part of diabetes care. Previous studies reported that not practicing handwashing leads to differences in capillary glucose concentrations [9]. Handwashing prior to testing blood glucose levels and administering insulin is recommended to obtain accurate results, avoid contamination of materials, and prevent infection at the injection site [25,26,27]. Due to the nature of the KCHS, which targets the general population, this study could not investigate diabetes-specific hand hygiene practices. Further studies are needed to investigate the hand hygiene practices that are required for blood glucose testing and insulin administration. 

Those who were educated about diabetes management reported frequent handwashing. Diabetes is a chronic, progressive disease; thus, adequate self-care and associated educational campaigns are essential [28]. Randomized controlled trials have found that conveyance of such educational information results in improved self-care and patient outcomes [29,30]. However, only 27.9% of people with diabetes reported being educated about diabetes management in this study. Therefore, healthcare providers must try to educate their patients to improve self-care practices, including the conveyance of information about hand hygiene. Greater than 68% of people with diabetes who were exposed to educational or public relations campaigns about handwashing reported more frequent handwashing. National campaigns for handwashing are widely performed in Korea [31], and various educational programs and materials have been developed, including lecture materials and visual tools. These results suggest that public handwashing campaigns will help improve the hand hygiene practices of people with diabetes. Further studies are recommended to assess the effects of specific hand hygiene educational materials, which are required to teach people with diabetes about self-care and hand hygiene practices.

This study has certain limitations. First, the KCHS did not collect detailed clinical data, such as age at diagnosis, admission experiences, and current diabetes status. Further studies are needed to investigate the hand hygiene practices of patients with diabetes in conjunction with their clinical status. Second, a social desirability bias could affect the responses regarding hand hygiene practices. Handwashing campaigns at the government and community levels have been widely carried out in Korea since 2005 [31], and most people reported being aware of the efficacy of handwashing in this study. Third, the KCHS focused on the community and did not include people with diabetes in general or convalescent hospitals. However, handwashing is recommended in both community and hospital settings, and community-based approaches may enhance the self-management of people with diabetes [3]. Fourth, this study included only people with diabetes who were aware of their condition due to use of the self-reported method, not blood tests, and could not distinguish between type 1 and 2 diabetes. However, assessing factors that result in individuals not performing appropriate hand hygiene, despite knowing that they have diabetes, would be important for education and management of diabetes regardless of the type. Finally, exposure to education and public campaigns was not specified, such as content and methods of education method. In previous studies, the effect of education on hand hygiene varied depending on the type, contents, and delivery method of education [32,33]. Therefore, if further studies define the type and content of education, it would be useful for constructing education programs.

## 5. Conclusions

Although nearly all study participants recognized that hand hygiene was able to prevent infectious diseases, handwashing rates were significantly lower in people with diabetes compared to an age- and gender-matched comparison group. Both demographic and diabetes-associated characteristics, such as being educated about diabetes management, treatment methods, and awareness of HbA1c level, were all associated with hand hygiene practices in people with diabetes. Educational programs about hand hygiene and diabetes management utilizing various treatment methods are needed to improve hand hygiene practices in patients with diabetes.

## Figures and Tables

**Table 1:** General characteristics of participants with and without diabetes.

Characteristics	With Diabetes (*n* = 22,920)	Without Diabetes (*n* = 22,920)
Gender		
Male	11,131 (48.6)	11,131 (48.6)
Female	11,789 (51.4)	11,789 (51.4)
Age (years)		
19–44	1131 (4.9)	1131 (4.9)
45-64	8850 (38.6)	8850 (38.6)
≥65	12,939 (56.5)	12,939 (56.5)
Marital status		
Single	523 (2.3)	566 (2.5)
With spouse	16,385 (71.6)	15,939 (69.6)
Divorced/separated/widowed	5990 (26.2)	6396 (27.9)
Education		
Uneducated	4981 (21.8)	4895 (21.4)
Elementary school	5910 (25.8)	6308 (27.6)
Middle school	3457 (15.1)	3591 (15.7)
High school	5296 (23.2)	5346 (23.4)
≥ College	3237 (14.2)	2744 (12.0)
Smoking		
Never	13,509 (58.9)	13,093 (57.1)
Former	5777 (25.2)	6091 (26.6)
Current	3634 (15.9)	3736 (16.3)
Alcohol drinking frequency		
None	9770 (42.6)	11,012 (48.1)
≤1 drink/month	4855 (21.2)	4560 (19.9)
≥2 drinks/month	8287 (36.2)	7341 (32.0)
Disease history, excluding diabetes		
No	9467 (41.3)	5003 (21.8)
Yes	13,453 (58.7)	17,917 (78.2)
Treatment for diabetes		
None (including nonpharmacologic methods)	2749 (12.0)	-
Oral hypoglycemic agent	18,329 (80.0)	-
Insulin	1839 (8.0)	-
Educated about diabetes management		
No	16,527 (72.1)	-
Yes	6393 (27.9)	-
Awareness of HbA1c level		
No	16,794 (73.3)	-
Yes	6125 (26.7)	-
Examined for complications during the past year		
No	11,983 (52.3)	-
Yes	10,937 (47.7)	-

Data are expressed as a number (%). HbA1c: hemoglobin A1c.

**Table 2:** Hand hygiene practices among people with and without diabetes.

Hand Hygiene Practices	With Diabetes	Without Diabetes	*p* Value
Awareness of handwashing efficacy, *n* (%)	22,276 (98.4)	22,348 (98.5)	0.412
aOR (95% CI) for handwashing ^1^	0.93 (0.80–1.09)	1.00	
Frequent handwashing			
Before eating, *n* (%)	19,318 (84.3)	19,723 (86.1)	< 0.001
aOR (95% CI) for handwashing ^1^	0.87 (0.83–0.92)	1.00	
After using the restroom, *n* (%)	18,876 (82.4)	19,218 (83.9)	0.001
aOR (95% CI) for handwashing ^1^	0.90 (0.85–0.94)	1.00	
After returning from the outdoors, *n* (%)	16,607 (72.5)	17,039 (74.3)	< 0.001
aOR (95% CI) for handwashing ^1^	0.91 (0.88–0.95)	1.00	
With soap or hand sanitizer, *n* (%)	14,687 (64.1)	14,950 (65.2)	0.010
aOR (95% CI) for handwashing ^1^	0.95 (0.91–0.98)	1.00	
Exposure to educational and public relations campaigns about handwashing, *n* (%)	15,705 (68.7)	15,927 (69.6)	0.043
aOR (95% CI) for handwashing ^1^	0.95 (0.91–0.99)	1.00	

aOR: adjusted odds ratio; CI: confidence interval. ^1^ Adjusted for age, sex, marital status, education, smoking, alcohol drinking frequency, and disease history.

**Table 3:** Factors associated with frequent handwashing among people with diabetes.

Characteristics	Before Eating		After Using the Restroom		After Returning from the Outdoors		With Soap or Hand Sanitizer	
	%	aOR (95% CI)	%	aOR (95% CI)	%	aOR (95% CI)	%	aOR (95% CI)
Gender								
Male	77.8	1.00	76.9	1.00	69.7	1.00	65.7	1.00
Female	90.5	3.21 (2.83–3.65)	87.6	2.86 (2.53–3.23)	75.0	1.63 (1.47–1.81)	62.6	1.24 (1.13–1.37)
Age (years)								
19–44	85.1	0.88 (0.72–1.09)	88.8	1.26 (1.00–1.57)	81.5	1.18 (0.99–1.42)	79.8	1.42 (1.19–1.69)
45–64	84.9	0.99 (0.91–1.08)	84.5	1.11 (1.01–1.21)	75.1	1.00 (0.93–1.08)	70.2	1.16 (1.08–1.24)
≥65	83.8	1.00	80.3	1.00	69.8	1.00	58.5	
Marital status								
Single	79.2	1.00	81.5	1.00	72.3	1.00	70.7	1.00
With spouse	84.1	1.26 (1.01–1.58)	82.7	1.17 (0.92–1.49)	73.8	1.21 (0.98–1.49)	66.6	1.13 (0.93–1.39)
Divorced/separated/widowed	85.2	1.05 (0.82–1.34)	81.7	1.00 (0.78–1.29)	69.1	1.04 (0.83–1.29)	57.3	1.02 (0.82–1.26)
Education								
Uneducated	84.0	1.00	78.3	1.00	65.3	1.00	49.6	1.00
Elementary school	84.2	1.26 (1.13–1.41)	80.7	1.38 (1.25–1.53)	70.0	1.26 (1.16–1.38)	59.8	1.40 (1.29–1.52)
Middle school	83.4	1.38 (1.21–1.59)	81.6	1.66 (1.46–1.88)	73.6	1.56 (1.39–1.74)	66.3	1.75 (1.58–1.94)
High school	84.3	1.57 (1.37–1.80)	85.3	2.23 (1.95–2.54)	77.1	1.82 (1.63–2.03)	73.6	2.31 (2.08–2.56)
≥ College	86.2	1.96 (1.66–2.31)	88.8	3.10 (2.62–3.66)	80.6	2.21 (1.92–2.54)	78.4	2.85 (2.50–3.24)
Smoking								
Never	89.2	1.37 (1.21–1.56)	86.7	1.53 (1.35–1.73)	75.0	1.23 (1.10–1.37)	63.3	0.98 (0.88–1.09)
Former	78.3	1.13 (1.02–1.26)	77.7	1.34 (1.21–1.48)	69.4	1.08 (0.99–1.19)	64.0	0.94 (0.86–1.03)
Current	76.9	1.00	74.8	1.00	68.7	1.00	67.0	1.00
Alcohol drinking frequency								
None	85.3	0.88 (0.80–0.97)	82.7	0.95 (0.87–1.05)	71.6	0.95 (0.88–1.03)	61.7	1.08 (1.01–1.16)
≤1 drink/month	86.6	0.94 (0.84–1.05)	85.2	1.00 (0.89–1.11)	75.0	0.98 (0.89–1.07)	65.8	1.01 (0.93–1.10)
≥2 drinks/month	81.3		80.1	1.00	72.2	1.00	66.6	1.00
Disease history, excluding diabetes								
No	84.0	1.00	82.1	1.00	72.4	1.00	65.8	1.00
Yes	84.4	0.90 (0.82–0.98)	82.4	0.96 (0.88–1.05)	72.5	1.03 (0.96–1.11)	63.6	1.03 (0.96–1.11)
Treatment for diabetes								
None (including nonpharmacologic methods)	84.5	1.19 (0.99–1.42)	84.2	1.30 (1.09–1.54)	75.3	1.36 (1.18–1.58)	68.3	1.33 (1.16–1.52)
Insulin	83.5	1.00	81.9	1.00	72.2	1.00	64.6	1.00
Oral hypoglycemic agent	84.3	1.12 (0.97–1.28)	82.1	1.12 (0.97–1.28)	72.1	1.15 (1.02–1.28)	63.4	1.12 (1.01–1.25)
Educated about diabetes management								
No	83.5	1.00	81.3	1.00	70.2	1.00	61.4	1.00
Yes	86.4	1.13 (1.03–1.23)	85.1	1.09 (1.01–1.19)	78.3	1.33 (1.23–1.43)	71.0	1.28 (1.20–1.37)
Awareness of HbA1c level								
No	83.3	1.00	80.4	1.00	69.9	1.00	60.3	1.00
Yes	86.9	1.15 (1.05–1.27)	87.8	1.29 (1.17–1.42)	79.5	1.21 (1.11–1.31)	74.5	1.23 (1.14–1.33)
Examined for complications during the past year								
No	83.4	1.00	80.6	1.00	70.0	1.00	61.1	1.00
Yes	85.3	1.06 (0.98–1.15)	84.3	1.13 (1.05–1.22)	75.2	1.14 (1.07–1.22)	67.4	1.12 (1.06–1.19)
Exposure to educational and public relations campaigns about handwashing								
No	78.4	1.00	75.0	1.00	63.8	1.00	54.5	1.00
Yes	87.0	1.68 (1.56–1.82)	85.8	1.68 (1.56–1.81)	76.5	1.55 (1.46–1.65)	68.5	1.46 (1.37–1.55)

aOR: adjusted odds ratio; CI: confidence interval.
